# Evaluation of matrix metalloproteinase type IV-collagenases in serum of patients with tumors of the central nervous system

**DOI:** 10.1007/s11060-016-2297-4

**Published:** 2016-10-18

**Authors:** Serena Ricci, Elia Guadagno, Dario Bruzzese, Marialaura Del Basso De Caro, Carmela Peca, Francesco G. Sgulò, Francesco Maiuri, Angelina Di Carlo

**Affiliations:** 1 Department of Medico-Surgical Sciences and Biotechnologies, “Sapienza” University of Rome, Corso della Repubblica 79, 04100 Latina, Italy; 2 Department of Translational Medical Science, University of Naples “Federico II”, Via S. Pansini 5, 80131 Naples, Italy; 3 Department of Advanced Biomorphological Sciences, University of Naples “Federico II”, Via S. Pansini 5, 80131 Naples, Italy; 4 Department of Public Health, University of Naples “Federico II”, Via S. Pansini 5, 80131 Naples, Italy; 5 Department of Neurosciences, Reproductive Sciences and Odontostomatology, University of Naples “Federico II”, Via S. Pansini 5, 80131 Naples, Italy

**Keywords:** Matrix metalloproteinases, Serum, Tumors, Central nervous system

## Abstract

The basement membrane collagen IV-degrading matrix metalloproteinases -2 and -9 (MMPs) are most often linked to the malignant phenotype of tumor cells by playing a critical role in invasion, metastasis, angiogenesis, and vasculogenesis. We verified the activity of these two MMPs in the sera of patients affected by brain tumors (20 gliomas, 28 meningiomas and 20 metastasis) by zymography. The sera of 25 healthy volunteers with no concomitant illnesses were used for controls. Zymography showed four dominant gelatinolytic bands of 240, 130, 92 (MMP-9) and 72 (MMP-2) kDa. No statistically significant variations of MMP-2 proteolytic activity between patients and healthy individuals were observed. On the contrary, MMP-9 (both monomeric and multimeric forms) lytic activities were significantly higher in tumors specimens compared to healthy controls (p < 0.001). Moreover, MMP-9 immunohistochemistry revealed: (1) a strong reactivity in neoplastic vessels of high-grade gliomas showing an inverse correlation with serum multimeric gelatinolytic activity; (2) a cytoplasmatic reactivity in meningiomas with a significantly increase in atypical meningioma compared with low-grade ones (p = 0.036); (3) a positive correlation between MMP-9 and Ki-67 (Sperman Rho coefficient r = 0.418 and p = 0.034). Our results suggest that serum and tissue MMP-9 might provide clinicians additional objective information in intracranial neoplasms. Finally, it should be possible to use MMP-9 as a target for new forms of therapy. Nevertheless, due to the small number of patients included in the study, the conclusion may not be transferable to the general population and therefore further evaluations are needed.

## Introduction

Tumors of the central nervous system (CNS) represent one of the most common causes of cancer death and account for about 1.3 % of all malignant cancer, with an incidence of 7 per 100,000 persons worldwide [1, 2]. CNS tumors consist of a heterogeneous group of neoplasms, including different variant of primary brain tumors (glial or non-glial, benign or malignant) and metastatic neoplasms [3, 4]. Metastatic brain tumors include malignant tumors that arise elsewhere in the body (such as the breast or lungs) and migrate to the brain, usually through the bloodstream. The number of primary and metastatic brain tumors is steadily climbing, whereas mortality rate for most tumor types have remained essentially unchanged. In particular, patients with high grade glioma usually have the worst prognosis with a median survival of 12 months even after surgical resection, radiation therapy and chemotherapy [5].

Actually, the main diagnostic tools for both primary and metastatic CNS tumors are the anamnestic neurological examination, the imaging tests, such as conventional magnetic resonance (MRI) and computerized tomography (CT) scan [2]. Advanced imaging techniques improve the neuro-radiological diagnostic accuracy; however, they expensive and lack of specificity, thus there is a pressing need of non-invasive methods to diagnose carcinoma of the CNS as well as for their management. Currently, except for rare germ cell tumors, there is no method to prospectively detect brain tumor until they have progressed to sympt stage. Thus, detection, definition and validation of new biomarkers for diagnosis, prognosis, disease monitoring, as well as therapeutic efficacy and tumor progression, are recognized as formidable challenges in oncologic research.

For long time basic cancer research has mainly focused on mutations in cancer cells that result in either gain or loss some functions of cells. However, more recent evidences characterize cancer development as the result of disrupted intra- and inter-cellular homeostatic regulation. Once the homeostatic balance has been lost and malignant transformation has occurred, micro-environmental processes, such as degradation of matrix components and host-tumor interactions, promote survival and growth of malignant cells [6]. Therefore, proteins and enzymes involved in degradation of extra-cellular matrix (ECM) have been shown to be essential for cancer progression, by providing tumor cells with access to vascular and lymphatic systems which support tumor growth and represent an escape route for further dissemination. Among all proteolytic enzymes potentially associated with tumor invasion, members of the matrix metallo-proteinases (MMPs) family are prime candidates, due to their ability to collectively degrade all components of the ECM and basement membranes [7–11]. In particular, the ability to degrade type IV collagen (gelatin), the major component of the ECM and basement membranes, is unique to gelatinase A (MMP-2) and gelatinase B (MMP-9) [9]. Both MMP-2 and MMP-9 are expressed in fibroblasts, leukocytes, macrophages and endothelial cells, and are involved in the mechanical removal of structural proteins in the extracellular matrix, but also in the regulation of multiple cellular functions including cell growth, apoptosis, angiogenesis and immune response, by cleaving growth factor-precursors, cell adhesion molecules, cell surface receptors and other bioactive proteins [12]. Nowadays, it is widely recognized that gelatinases participate to the aetiology of a plethora of normal biological and non-tumoral pathological processes, such as autoimmune diseases, cardiometabolic diseases, neurological disorders, breakdown of blood-brain-barrier, skin ulceration etc. [13]. They are also important in the formation of the complex microenvironment which (1) promotes malignant transformation in early steps of tumor evolution, (2) stimulates cell proliferation and modulate angiogenesis and vasculogenesis in late steps of tumor growth [13]. High expression of MMP-2 and MMP-9 has been often associated to malignant phenotype of many tumor types, with positive correlation with tumor grading and/or staging [14], thus, they obtained a deep interest as new potential biomarkers.

In the present study, we determined MMP-2 and MMP-9 activity in sera from patients with intracranial carcinoma, by using gelatin zymography, in order to analyze the pattern of gelatinolytic activity and to verify whether they may have potential as non-invasive biomarkers in providing useful clinical information.

## Materials and methods

### Study design

This was a unicentre observational study. All patients affected by intracranial neoplasms treated at the Neurosurgery Unit of the University of Naples “Federico II” in one year were potentially eligible. Patients were excluded if (1) they had other concomitant illnesses; (2) they had evidence of recent intracranial hemorrhage; (3) they had history of intracranial abscess; (4) they did not have a definitive diagnosis at the end of diagnostic work-up. MMP-2 and -9 were evaluated in serum by substrate gelatin zymography; MMP-9 tissue expression was evaluated by immunohistochemistry in surgical specimens of glioma and meningioma, not in metastases. In all meningiomas also Ki-67 and progesterone receptor (PR) stainings were performed. The protocol of this study was approved by the Hospital Ethics Committee of the University of Naples “Federico II” and written informed consent was obtained from all individuals before being included in the study.

### Patient population

A total of 68 patients with intracranial tumors were evaluated. Diagnosis of tumors was made by usual clinical criteria and confirmed post-operatively by histopathological findings according to the latest WHO classification of tumors of the central nervous system [3, 4]. The age of patients was between 17 and 87 years with a median age of 64 ± 15.2 and there were 37 males and 31 females. The study included 20 gliomas, 28 meningiomas and 20 metastases. The clinical-pathological characteristics are listed in Tables 1, 2 and 3. Twenty-five healthy volunteers with no concomitant illnesses were used as controls. The age of healthy volunteers was between 47 and 85 years (62 ± 10.3) and there were 11 males and 14 females. Healthy volunteers gave their permission verbally. The subjects in the controls had no sign of infections, gastrointestinal, hepatic or renal disease, nor tumors or immunological disease. The values of basic laboratory parameters of these participants were within the references limits.

**Table 1:** Serum MMP-2 and MMP-9 levels and tissue MMP-9 reactivity in glioma patients

Case	Sex	Age	Diagnosis	Localization	WHO Grade	Serum MMPs (vol × 10^−3^)				Tissue MMP-9	
						MMP-9 (240 kDa)	MMP-9 (130 kDa)	MMP-9 (92 kDa)	MMP-2 (72 kDa)	Neoplastic vessels	Neoplastic glial cells
1	M	43	A	Cerebellum	I	nd	nd	243	574	0	0
2	F	17	A	Encephalon	I	207	415	1482	249	0	0
3	M	39	G	Cerebellum	I	106	50	1243	576	0	0
4	F	32	O	Left temporal lobe	II	248	113	1765	942	0	+++
5	M	40	O	Left frontal lobe	II	62	58	1051	547	0	+++
6	M	49	AO	Right frontal lobe	III	103	123	1262	410	0	+++
7	F	82	AO	Right temporal lobe	III	342	214	1684	893	0	++
8	F	73	AO	Left frontal lobe	III	185	102	1262	359	0	++
9	M	73	AO	Encephalon	III	110	61	708	310	0	++
10	M	68	GMB	Left temporal lobe	IV	88	38	1015	460	++	0
11	M	56	GMB	Left temporal lobe	IV	111	115	1394	493	++	++
12	M	48	GMB	Left frontal lobe	IV	47	nd	667	464	+++	+
13	M	62	GMB	Right temporal lobe	IV	140	nd	1190	566	++	+
14	F	76	GMB	Right parietal lobe	IV	82	61	892	540	++	++
15	M	77	GMB	Encephalon	IV	117	nd	1096	392	Not done	Not done
16	M	75	GMB	Right parietal lobe	IV	194	96	1408	205	Not done	Not done
17	M	58	GMB	Left temporal lobe	IV	203	nd	1361	1026	0	+
18	F	63	GMB	Right temporal lobe	IV	101	nd	1130	526	++	++
19	M	78	GMB	Right temporal lobe	IV	162	61	470	70	0	+
20	F	45	GMB	Left frontal lobe	IV	77	nd	1117	308	Not done	Not done

*A* astrocytoma, *G* ganglioglioma, *O* oligodendroglioma, *AO* anaplastic oligodendroglioma, *GMB* glioblastoma multiforme, *MMP* matrix-metalloproteinase, *nd* not detectable, *0* no signal, + <10 %, ++ 10–30 %, +++ 30 %

**Table 2:** Serum MMP-2 and MMP-9 levels and tissue Ki-67, PR and MMP-9 reactivities in meningioma patients

Case	Sex	Age	Diagnosis	Localization	WHO Grade	Serum MMPs (vol × 10^−3^)				Ki-67 (%) (LI)	PR (%) (LI)	Tissue MMP-9
						MMP-9 (240 kDa)	MMP-9 (130 kDa)	MMP-9 (92 kDa)	MMP-2 (72 kDa)			
21	M	65	Syncytial	Planum etmoidale	I	62	nd	437	575	2	90	0
22	F	50	Secretory	Left parietal lobe	I	160	149	1318	196	6	<10	0
23	F	70	Microcystic	Encephalon	I	93	60	1032	399	2	<10	0
24	F	54	Psammomatous	Spinal (D8–D9)	I	58	111	1185	227	3	50	0
25	F	71	Psammomatous	Left tentorium	I	199	107	1008	290	<1	<10	0
26	M	36	Psammomatous	Encephalon	I	205	145	1494	1205	1–2	80	+
27	F	45	Fibroblastic	Left ventricular trine	I	90	52	1046	916	3	80	+++
28	F	75	Transitional	Planum etmoidale	I	233	222	1217	470	<3	70	0
29	F	68	Transitional	Left parietal lobe	I	56	nd	665	399	2	<10	+++
30	F	59	Transitional	Cerebellum	I	183	247	1425	1083	3	10	++
31	F	54	Transitional	Right anterior cranial fossa	I	77	130	1000	565	2	30	+
32	F	47	Transitional	Right spheno-orbital	I	74	nd	525	587	4	<1	+
33	F	38	Transitional	Planum etmoidale	I	104	78	1336	318	2–3	90	+
34	F	49	Transitional	Sphenoid	I	49	nd	769	396	2	70	+
35	M	66	Transitional	Parasagittal	I	131	67	1092	643	<1	<1	0
36	F	36	Transitional	Planum etmoidale	I	44	nd	750	202	<1	<1	+
37	M	76	Atypical	Right parietal convexity	II	190	nd	1099	618	5	80	+
38	F	72	Atypical	Dorsal (D8-D9)	II	223	109	1362	546	13	<10	+++
39	M	70	Atypical	Right frontal convexity	II	157	138	1563	754	6	50	0
40	F	58	Atypical	Left frontal convexity	II	33	nd	572	569	7	50	+++
41	M	84	Atypical	Left parietal convexity	II	148	106	1298	712	7–8	<10	++
42	M	64	Atypical	Parasagittal	II	58	nd	803	461	10	<10	+++
43	M	77	Atypical	Parasagittal	II	73	74	1248	753	10–12	<10	+
44	M	80	Atypical	Left frontal convexity	II	72	46	551	381	6–7	30	+++
45	F	50	Atypical	Right petrous	II	114	124	1238	324	10	<10	+++
46	F	82	Atypical	Right anterior cranial fossa	II	156	54	1186	794	10	75	+
47	F	73	Atypical	Left parietal lobe	II	85	nd	937	511	6–7	50	0
48	M	51	Atypical	Parasagittal	II	nd	nd	756	48	50	40	+

*Ki-*67 proliferative marker, *PR* progesteron receptor, *LI* labelling index, *MMP* matrix-metalloproteinase, *nd* not detectable, *0* no signal, + <10 %, ++ 10–30 %, +++ 30 %

**Table 3:** Serum MMP-2 and MMP-9 levels in brain metastasis patients

Case	Sex	Age	Diagnosis	Localization	Serum MMPs (vol × 10^−3^)			
					MMP-9 (240 kDa)	MMP-9 (130 kDa)	MMP-9 (92 kDa)	MMP-2 (72 kDa)
49	M	64	Melanoma	Left temporal lobe	212	nd	1233	755
50	M	61	Melanoma	Right frontal lobe	160	67	1426	703
51	M	66	Melanoma	Cerebellum	208	104	1717	330
52	M	50	Melanoma	Frontal lobe	100	45	940	384
53	F	58	Melanoma	Encephalon, NOS	198	318	1183	540
54	F	52	Melanoma	Left frontal lobe	145	111	1450	1228
55	M	32	Melanoma	Parietal lobe	149	233	1194	334
56	M	77	Melanoma	Encephalon, NOS	306	719	2040	1132
57	M	67	Melanoma	Right temporal lobe	305	217	1531	470
58	M	80	Melanoma	Right occipital lobe	89	nd	556	353
59	F	87	NSCLC	Right cerebellum	254	nd	1202	518
60	M	57	NSCLC	Left occipital lobe	361	195	1672	485
61	M	48	NSCLC	Encephalon, NOS	185	72	1473	781
62	M	72	NSCLC	Left frontal lobe	140	nd	1277	535
63	M	55	NSCLC	Left cerebellum	403	235	1740	1012
64	M	71	SCLC	Cerebellum	122	147	1217	237
65	F	67	Breast Ca	--	80	nd	1097	634
66	F	77	Colon Ca	Cerebellum	121	51	1066	334
67	M	73	Kidney Ca	Left frontal lobe	117	103	1077	213
68	F	77	Urogenital Ca	Left frontal lobe	135	164	1143	125

*NSCLC* non-small lung carcinoma, *SCLC* small lung carcinoma, *MMP* matrix-metalloproteinase, *nd* not detectable

### Serum samples

Peripheral venous blood samples were collected preoperatively. Native serum was prepared using plastic tubes without coagulation accelerators, to prevent the release of gelatinases during platelet activation. Tubes were centrifuged at 1600 g for 10 min, 30 min after blood collection. For each sample, determination of protein concentration was performed using the method of Bradford [15]. Sera were aliquoted and stored at −20 °C until used. Each aliquot was used only once in order to prevent enzyme activation due to freeze-thawing processes.

### Materials

Gelatinase A and gelatinase B were purchased from Hoffmann-La Roche Ltd (Basel, Switzerland). Calcium chloride (CaCl_2_) glycerol, gelatin, ethylenediaminetetraacetic (EDTA), Triton X-100, phenylmethylsulphonyl fluride (PMSF) were from Sigma Chemical Co. (St. Louis, MO, USA). Ki-67 antibody (MIB1) from Dako (Milano, Italy); progesterone receptor antibody (1E2) from Ventana Medical Systems Inc. (Tucson, AZ, USA); and MMP-2, MMP-9 antibodies from Sigma Chemical Co. (St. Louis, MO, USA). All other reagents were available from commercial sources.

### Gelatin zymography

Gelatinolytic activity was performed as previously described [16]. Briefly, total protein (25 µg) of each sample was mixed with sample buffer (10 mM Tris–HCl pH 6.8, 12.5 % SDS, 5 % sucrose, 0.1 % bromophenol blue) and applied directly without prior heating or reduction to 7.5 % (w/v) acrylamide gels containing 0.3 % (w/v) of gelatin. After removal of SDS from the gel by incubation in 2.5 % (v/v) Triton X-100 for 1 h, the gels were incubated at 37 °C for 18 h in 50 mM Tris–HCl pH 7.6 containing 0.2 M NaCl, 5 mM CaCl2, and 0.02 % (w/v) Brij 35. Gels were stained for 1 h in 30 % methanol, 10 % glacial acetic acid containing 0.5 % (w/v) Coomassie Brilliant Blue G 250 and destained in the same solution without dye for several hours. The gelatinolytic activity of each collagenase was evident as a clear band against the blue background of stained gelatin. The molecular size of bands displaying enzymatic activity were identified by comparison with prestained standard protein, as well as with purified gelatinase A or B. To normalize the possible difference between zymograms an internal serum sample from a patient was incorporated in every gel. Control gels contained either of the MMP selective inhibitors, 20 mM EDTA or 10 mM 1,10 phenanthroline, in the MMP incubation buffer to confirm that lysis band was the results of MMPs. Furthermore, the character of proteolytic bands was analyzed by incubating the identical zymograms in 0.1 mg/ml of PMSF, a serine protease inhibitor; or 2 mM Pefabloc, an irreversible serine protease inhibitor. Following zymography, the degree of gelatin digestion was quantified as previously described [16]. Briefly, we used an image analysis software (ImageQuant TL, Amersham Bioscience, Chicago, IL, USA) according to the manufacturer’s specifications. The image of the gel was inverted to reveal dark bands on a white background. The molecular weight, volume and background of each band were determined. The relative amounts of the different forms of gelatinases were expressed as the integrated density ×10^−3^ (volume) of all the pixels above the background of each band.

### Immunohistochemistry

In 45 surgical resected specimens, consisting of 17 glial tumors and 28 meningiomas, immunohistochemical evaluation with MMP-9 antibody was performed. All tissues were fixed in 10 % neutral formalin for 24 h at room temperature, embedded in paraffin at 55 °C and cut firstly in 4 μm thick sections that were stained with conventional routine hematoxylin and eosin stain used for diagnostic histological examination; afterwards, additional 4 μm thick sections were used for immunohistochemistry. After dewaxing in xylene, rehydratation in alcohol decreasing scale and heat-induced epitope retrieval in Tris EDTA buffer (pH 9), endogen peroxidase block with 3 % H_2_O_2_ followed. Sections were then incubated for 90 min, at room temperature, with anti MMP-9 monoclonal primary antibody (Abcam EP 1254, rabbit), at 1:100 dilution. A streptavidin-horseradish peroxidase detection system and subsequent chromogen reaction with diaminobenzidine (Dako) were applied. Counterstaining was performed using Harris hematoxylin. Sections of tissue form gastric adenocarcinoma were used as positive control whereas the negative control was a section of glioma and one of meningioma stained with the secondary antibody alone. A different interpretation of MMP-9 signal was necessary in the two groups of malignancies: in gliomas the site and the percent (0: no signal; +: <10 %; ++: 10–30 %; +++: >30 %) of reactivity were evaluated, while meningiomas that usually display a more homogeneous cellular composition, only the percentage of reactivity was considered (0: no signal; +: <10 %; ++: 10–30 %; +++: >30 %). The immunostaining was evaluated separately by three different pathologists who ignored any clinical information and, in case of discordance, a second observation was made to a multi-head microscope, in order to reach agreement. For meningiomas, Ki-67 (MIB1, Dako) and PR (1E2, Ventana) stainings were performed automatically with prediluted antibodies. Ki-67 Label Index (LI) count was performed by taking the average on five representative fields of neoplastic cells in hot spot areas. A cut-off point to consider low and high Ki-67 expression was set at 4 %. An evaluation of percent of nuclear immunoreactivity for PR amongst the neoplastic cells was carried out.

### Statistical analysis

All statistical analyses were performed with R statistical platform (vers. 3.2.3, the R Foundation for Statistical Computing). Quantitative variables were described with median and range and compared between groups using the non parametric Kruskall Wallis test followed by Mann Whitney U test for pairwise comparisons. Correlation among variables were assessed using the non parametric Spearman coefficient. All statistical tests were two sided with a significance level set at 0.05. No correction for multiple comparisons was undertaken.

## Results

**Fig. 1:**
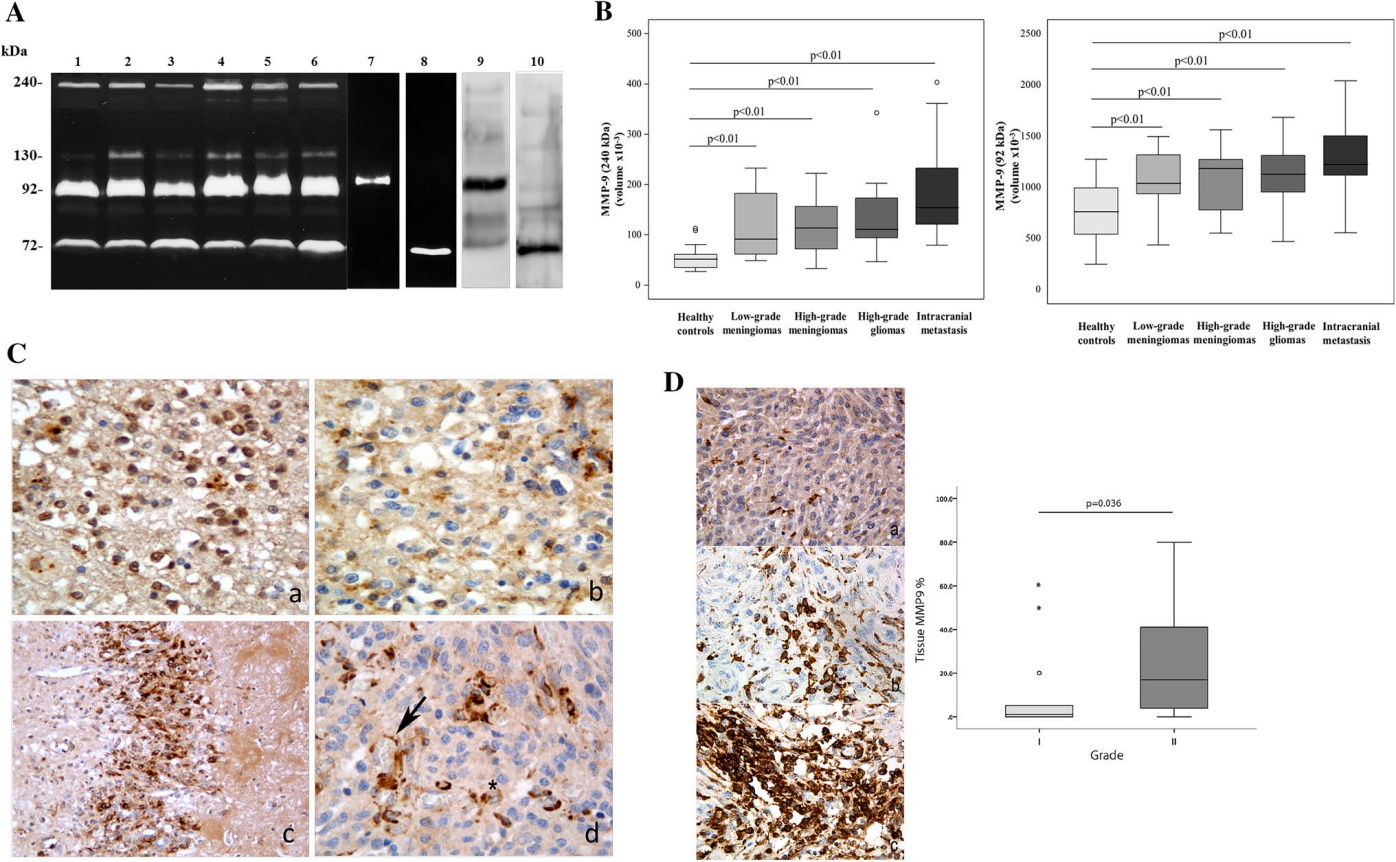
Panel **a** Representative gelatin zymography of serum (*lanes 1–6*), purified gelatinases, and Western blotting. Molecular weight are shown on the *left. Lane 1* healthy subject, *lane 2* glioblastoma multiforme (patient 11); *lane 3* oligodendroglioma (patient 5), *lane 4* intracranial metastasis from melanoma (patient 57), *lane 5* low-grade meningioma (patient 25), *lane 6* high-grade meningioma (patient 43), *lane 7* purified gelatinase B (MMP-9, 92 kDa) 20 µU, *lane 8* purified gelatinase A (MMP-2, 72 kDa) 120 mU, *lane 9* Western blotting of MMP-9 (92 kDa); *lane 10*: Western blotting of MMP-2 (72 kDa). Panel **b** *Box plot* showing the distribution of multimeric form (240 kDa) and monomeric form (92 kDa) of serum MMP-9 in healthy controls, low-grade meningioma (grade I), high-grade meningioma (grade II), high-grade glioma (GB) and intracranial metastases. Value of integrated density are expressed as volume ×10^−3^. Data are shown as median (*horizontal line* in the box), Q1 and Q3 (*border* of the box) and min and max (*whiskers* outside the box). *Dot* represent outliers values (i.e., data points below Q1 −1.5 × IQR or above Q3 +1.5 × IQR). Q1 = 25th percentile; Q3 = 75th percentile; IQR (interquartile range) = Q3–Q1. Panel **c** Immunohistochemestry against MMP-9 in gliomas; (a) grade II glioma (oligodendroglioma): diffuse cytoplasmic and nuclear immunoreactivity in neoplastic cells (400× magnification); (b) grade III glioma (anaplastic oligodendroglioma): strong cytoplasmic signal inside tumor cells (400× magnification); (c) grade IV glioma (glioblastoma): heavy immunoreactivity in neoplastic endothelial cells lying at the interface of tumor-cerebral parenchyma (200× magnification); (d) grade IV glioma (glioblastoma): deep immunoreactivity in neoplastic vessel endothelial cells and occasional cytoplasmic signal within the tumor (400× magnification). Panel **d** Immunohistochemestry against MMP-9 in meningiomas (400× magnification) and box *plot* showing MMP-9 staining percentage in low- and high-grade meningiomas. (a) grade I meningioma: cytoplasmic immunoreaction in less than 10 % of neoplastic cells (b) grade II meningioma: cytoplasmic immunoreaction in 10–30 % of neoplastic cells (c) grade II meningioma: cytoplasmic immunoreaction in more than 30 % of neoplastic cells

To investigate the gelatinolytic activity present in the serum, substrate gel zymography was performed. This method allows the detection of the metalloproteinases that exhibit gelatinolytic activity. Representative zymography results are shown in Fig. 1 panel a. Polyacrylamide gels were evaluated for the presence of clear zone representing degradation of gelatin by proteolysis. The nature of lytic bands was confirmed by inhibition assay with a selective inhibitors of serine proteases and with selective inhibitors of MMPs (data not shown). Moreover, the immunological detection of lytic bands has been confirmed by performing Western blotting with antibodies against MMP-2 and MMP-9 (Fig. 1, panel a, lanes 9, 10). In the sera of all patients, the gels revealed the existence of four clear zones representing degradation of gelatin by proteolysis migrating at approximately 240, 130, 92 kDa (MMP-9) and 72 kDa (MMP-2), respectively. Comparison of these gelatinolytic bands with prestained standard protein and purified gelatinase A (MMP-2) and gelatinase B (MMP-9) clearly identified the MMP constituting bands as gelatinase A (MMP-2; 72 kDa) (Fig. 1, panel a, lane 8) and gelatinase B (MMP-9; 92 kDa) (Fig. 1, panel a lane 7). The clear zones with molecular weight >92 kDa might represent complexes of MMPs that are not dissociated in zymography. In fact, MMP-9 can be associated with a 25-kDa protein (lipocalin) giving a band at ~125 kDa [17, 18] and can form a complex with its endogenous inhibitors TIMP-1 giving a band at ~140 kDa [19]. Furthermore, MMP-9 can form dimer or multidimer giving a lytic band at approximately 240 kDa [19]. Following gelatin zymography, the proteolytic bands were subjected to densitometric analysis and the data, normalized to an internal serum standard, were expressed as the integrated density of all pixels of each band (volume ×10^− 3^). A summary of expression patterns of proteinases in glioma, meningioma, and brain metastasis specimens is shown in Tables 1, 2 and 3.

Considering the volume average of each individual band, we observed that enzymatic activity of both monomeric and multimeric forms of MMP-9 are significantly higher in high-grade glioma, in low- and high-grade meningioma samples as well as in metastasis specimens compared to healthy individuals (p < 0.001) (Fig. 1, panel b, and Table 4). No statistically significant variations of MMP-2 proteolytical activity between patients and healthy individuals have been observed.

**Table 4:** Median values and range of MMP-2 and the three MMP-9 forms gelatinolytic activities in sera from glioma, meningioma and intracranial metastasis patients

	MMP-9 (240 kDa)	MMP-9 (130 kDa)	MMP-9 (92 kDa)	MMP-2 (72 kDa)
Healthy controls	51.7 [27; 112.5]	61.4 [48.8; 90.4]	762.6 [248.7; 1274.8]	438.9 [201.9; 1117.6]
High grade gliomas	111.2 [47; 342.5]*	98.8 [38.4; 213.8]	1129.9 [470.2; 1684.2]*	459.6 [69.8; 1025.7]
Low grade meningiomas	91.5 [48.6; 232.9]*	120.2 [51.6; 247.2]	1038.9 [436.6; 1493.9]*	490.3 [195.8; 1205.4]
High grade meningiomas	113.7 [32.8; 222.6]*	106 [45.6; 137.7]	1185.9 [550.8; 1562.6]*	617.8 [324.4; 794.4]
Intracranial metastasis	154.7 [79.6; 403.1]*	146.8 [45.2; 719.1]	1224.9 [556.2; 2039.7]*	501.9 [125.1; 1228.4]

*Significantly different from controls (p < 0.01)

A further objective of this study was to correlate expression of serum MMP-9 with the expression of the same protein in the tumor tissue (the putative source of the biomarker). To address this aim, glioma and meningioma tissues were subjected to immunohistochemistry with MMP-9 antibody. Among glial neoplasms, MMP-9 immunohistochemical expression (Table 1) was absent in vessels and diffusely present in glioma cells, both cytoplasmic and nuclear, when of grade II; in grade III gliomas the signal was weaker only cytoplasmic in glioma cells and almost lacking in neoplastic vessels, were observable; in glioblastomas (grade IV), instead, a strong reactivity was detected in neoplastic vesels in comparison with gliomatous cells that were almost completely silent. Two cases of pylocitic astrocytoma and a ganglioglioma (grade I) did not reveal any signal (Fig. 1 panel c, and Table 1). In particular, we observed a strong positivity in 6 out 8 samples (75 %) in neoplastic vessels of glioblastoma tissue specimens and a difference compared with lower grade glioma specimens (p = 0.002); however because of the small number of samples this difference may be not statistically significant. Moreover, we identified an inverse correlation between MMP-9 tissue expression in the endothelial cells of neoplastic vessels and serum multimeric MMP-9 gelatinolytic activity (240 kDa band) with a Sperman Rho of r = −0.683 and p = 0.062 values. As it concerns meningiomas, the staining revealed: (a) mainly cytoplasmic MMP-9 reactivity and a strong signal in atypical meningiomas compared with low-grade ones (p = 0.036) (Fig. 1, panel d, and Table 2); (b) Ki-67 index was more expressed in grade II meningioma compared with grade I specimens (p < 0.001), whereas progesterone receptor (PR) did not correlated with the tumor grading (p = 0.257). Finally, we found a positive correlation between MMP-9 and Ki-67 with a Sperman Rho of r = 0.418 and p = 0.034 values.

## Discussion

Since most symptoms associated with primary and/or secondary brain tumors are also common to other diseases, the decision whether -or not- to investigate for a possible tumor is difficult. In fact, neuro-imaging techniques (the gold standard methods in evaluation for brain tumors) are relatively expensive and may identify innocent lesions. Moreover, diagnosis must be confirmed with histopathological examination of tissue samples [20]. Thus, there is a great interest in identifying reliable blood biomarkers that could support the management of brain tumors, e.g. facilitating neuro-radiological differential diagnosis at initial presentation, planning of surgical interventions and/or monitoring of the disease course [21, 22]. The role of MMPs has been studied extensively in number tumors of diverse origins, however, to our best knowledge little is known about their role in intracranial tumors. In this preliminary study, we measured gelatinolytic levels of serum forms of MMPs by zymography, and tissue expression of MMP-9 by immunohistochemistry. The zymographic tests have some advantages over immunological assay such as lower cost, a more rapid time of execution and possibility of simultaneously detecting multiple forms of the same enzyme. Our results showed that MMP-9 (92 and 240 kDa) is significantly increased in the sera from patients with CNS tumors compared to healthy individuals and differences in MMP-9 tissue expression have been underlined between glioblastoma and low-grade glioma specimens. Nevertheless, no relevant differences of MMP-9 lytic activities have been observed between low-grade and high-grade specimens. By immunoenzyme method (ELISA), *Hormigo* et al. observed that the levels of MMP-9 were higher in the sera samples of patients with high-grade glioma after surgery, while the MMP-9 concentrations were significantly lower in glioblastoma patients with no radiographic evidence of disease in comparison to the subjects with active tumor [23]. Anyway, MMP-9 increases following brain surgery, suggesting that increases in the serum level of this protein may be associated with brain inflammation and breakdown of blood brain barrier rather than be a true measure of tumor burden [23]. Vice versa, *Iwamoto* et al., in a larger cohort of glioma patients, observed no statistically significant association between levels of serum MMP-9 and radiographic disease status in both low- and high-grade glioma as well as between different type of CNS tumors [24]. On the contrary, immunohistochemical detection of MMP-9 in neoplastic tissues revealed a significantly different protein localization and distribution between low- and high-grade specimens. The most evident findings was, indeed, the de-localization of the MMP-9 signal from glioma cells (nucleus and cytoplasm) to endothelial cells of neoplastic blood vessels, which is directly related to tumor malignancy. Consistently, previous immunohistochemical and *in situ* hybridization studies had demonstrated that, in high-grade glioma, MMP-9 expression is mainly confined to perivascular regions at the infiltrating borders of the tumor and, in most of cases, to endothelial cells, with an intimate association with tumor malignant behavior [25, 26]. These observations may suggest differential regulation and utilization of MMP-9 during the progression of glial tumors, from low-grade to high-grade neoplasms, with a primary role of MMP-9 in tumor neovascularization. The up-regulation of MMP-9 in endothelial cells, in fact, contributes to angiogenesis initiated by these cells that, under specific angiogenic stimuli, degrade the basement membrane surrounding their vessel and migrate through the ECM into the surrounding tissue [27]. The interpretation of immunohistochemical results was conducted separately for gliomas and meningiomas because of their extremely different biological behavior.

Meningiomas encompass a large group of neoplasms with mainly benign behavior. Features of invasiveness and metastasis, defined as “brain-invasive meningiomas”, are generally typical of grade II meningiomas (atypical meningiomas). Thus, a better understanding of these invasive mechanisms could lay the groundwork for the development of more efficient therapeutic strategies. The comparative evaluation of MMP-9 activity levels in serum from low-grade and high-grade meningioma patients did not reveal significant differences, despite the positive predictive value of MMP-9 (92 and 240 kDa forms) in determining the presence or absence of brain tumor compared to control subjects. To our best knowledge, there are no previous evidences in evaluation of MMP-9 expression and/or activity in serum or plasma from meningioma patients. In a pilot study *Smith ER* et al. observed an increase of MMP-9 levels in urine from primary brain cancer patients which may be predictor of the presence or absence of tumor but without discriminating between tumor types or grades [28]. The data shown here indicate that tissue MMP-9 expression is significantly increased in atypical meningiomas compared to lower grade specimens, and is positively correlated with Ki-67 index levels. These results are in keeping with the data of other authors who observed a deeper expression of MMP-9 in high-grade meningiomas, accompanied by a positive correlation with tumor invasion and recurrence [29–32].

Our results suggest that the dosage of MMPs in the sera of CNS tumor patients and the immunohistochemical evaluation of MMP-9 and its correlation with Ki-67 only in meningiomas, might provide clinicians additional objective information on intracranial neoplasms. Finally, it should be possible to use MMP-9 as a target for new forms of therapy. Nevertheless, due to the small number of patients included in the study, the conclusion may not be transferable to the general population and therefore deserves further evaluation.
